# Supplementary material for: The Nicotine Metabolite Ratio and Response to Smoking Cessation Treatment Among People Living with HIV Who Smoke in South Africa
Source: Int J Environ Res Public Health. 2025 Jun 30;22(7):1040. doi: 10.3390/ijerph22071040 (PMC12294352; doi:10.3390/ijerph22071040)
Supplement: Supplementary file 1 [file ijerph-22-01040-s001.zip › ijerph-3676455-supplementary.pdf]

SUPPLEMENTAL MATERIALS

Table S1: Characteristics of the intent-to-treat population and of the subgroup included in NMR analysis

	**Total (n = 561)**	**NMR (n = 446)**	**Excluded (n = 115)**	**p-value**
Treatment arm				
BC	280 (50)	220 (49)	60 (52)	0.79
BC+cNRT	281 (50)	226 (51)	55 (48)	
Socio-demographic				
Gender				
Female	123 (22)	98 (22)	25 (22)	
Male	438 (78)	348 (78)	90 (78)	1.00
Age, median (IQR)	38 (31,46)	38 (31,45)	37 (31,46)	0.56
Age				
<30	116 (21)	88 (20)	28 (24)	0.77
30-35	115 (20)	91 (20)	24 (21)	
36-45	188 (34)	155 (35)	33 (29)	
>45	141 (25)	111 (25)	30 (26)	
Schooling				
<12th grade	472 (84)	375 (84)	97 (84)	0.90
≥ 12th grade	89 (16)	71 (16)	18 (16)	
Employment				
Unemployed	416 (74)	329 (73)	87 (76)	0.88
Employed	145 (26)	117 (26)	28 (24)	
Total monthly family income				
≤ 1000 R	234 (42)	184 (41)	50 (43)	0.87
>1000 R	326 (58)	261 (59)	65 (57)	
Tobacco use				
Heaviness of Smoking Index				
Low	144 (26)	108 (24)	36 (31)	0.34
Moderate/high	416 (74)	337 (76)	79 (69)	
Cigarettes per day				
<10 cigarettes	277 (49)	210 (47)	67 (58)	0.15
≥10 cigarettes	284 (51)	236 (53)	48 (42)	
Quit attempt in the past year				
No	213 (38)	163 (37)	50 (43)	0.39
Yes	348 (62)	283 (63)	65 (57)	
Exposed to second-hand smoke at home				
No	378 (68)	300 (68)	78 (68)	1.00
Yes	181 (32)	144 (32)	37 (32)	
Exhaled breath CO (ppm) median (IQR)	15 (9, 22)	15 (9,22)	13 (8,19)	0.05
Smokescreen baseline analysis				
Light	356 (70)	280 (69)	76 (72)	0.45
Moderate	89 (17)	68 (17)	21 (20)	
Heavy	65 (13)	56 (14)	9 (8)	
Other substance use				
Alcohol consumption				
No alcohol misuse	90 (23)	66 (22)	24 (28)	0.42
Alcohol misuse	302 (77)	239 (78)	63 (72)	

Current marijuana use				
No	179 (60)	137 (58)	42 (68)	0.19
Yes	120 (40)	100 (42)	20 (32)	
Clinical characteristics				
Current CD4+ T cell count				
<200 cells/μL	85 (25)	69 (25)	16 (29)	0.58
200-500 cells/μL	159 (48)	136 (49)	23 (42)	
>500 cells/μL	88 (27)	72 (26)	16 (29)	
Current Viral Load				
< 200 copies/mL	22 (25)	19 (26)	3 (23)	0.79
>200 copies/mL	65 (75)	55 (74)	10 (77)	
Current TB				
No	539 (96)	427 (96)	112 (97)	1.00
Yes	21 (4)	18 (4)	3 (3)	
Current cough				
No	338 (60)	271 (61)	67 (58)	0.82
Yes	223 (40)	175 (39)	48 (42)	
Body mass index				
Low (<18.5)	176 (32)	151 (32)	35 (30)	0.82
Normal (18.5 - 25)	313 (56)	250 (56)	63 (55)	
High (>25)	69 (12)	52 (12)	17 (15)	

COppm = carbon monoxide parts per million; BC = behavioral counseling; BC+cNRT = behavioral counseling plus combination nicotine replacement therapy; NMR = nicotine metabolite ratio

Tables S2: Multivariable modified Poisson regression of smoking abstinence at 6 months after controlling for known sources of NMR variation

	RR[a] (95% CI)	p-value
NMR (Ref. slow)	0.81 (0.45, 1.46)	0.48
Gender (Ref. male)	1.35 (0.74, 2.47)	0.32
Cigarettes/day (Ref. <10 cigarettes)	0.85 (0.51, 1.63)	0.75
Efavirenz use (Ref. no)	0.74 (0.74, 3.61)	0.22
Body mass index (Ref. low)		
Normal (18.5 – 25)	0.91 (0.50, 1.62)	0.74
High (>25)	1.60 (0.73, 3.51)	0.24

Table S3. Modification of the effect of NMR on smoking abstinence at 6 months by treatment arm

	Slow metabolizers	Normal metabolizers	NMR within strata of gender
		RR (95% CI)	RR (95% CI)
Gender			
Male	REF	0.91 (0.43, 1.91)	0.91 (0.43, 1.91)
Female	1.69 (0.90, 3.19)	1.07 (0.33, 3.51)	0.63 (0.18, 2.22)

Measure of effect modification on the multiplicative scale: RR (95% CI): 0.70 (0.16, 2.99); p = 0.63

Measure of effect modification on the additive scale: RERI (95% CI): -0.53 (-2.23, 1.18); p = 0.73

Table S4: Linear regression examining the association between NMR and secondary outcomes

	Change in exhaled Breath CO		Change in rine Cotinine		Change in Nicotine Withdrawal	
	β (95% CI)	*p* value	*β* (95% CI)	*p* value	*β* (95% CI)	*p* value
NMR (ref. slow s)	−1.39(−4.5,1.76)	0.39	0.98 (−1.18, 3.15)	0.75	1.01(−1.91, 3.93)	0.49

COppm = carbon monoxide; NMR = nicotine metabolite ratio; 95% CI = 95% confidence interval; β: Beta coefficient
